# Error‐Related Brain Potentials as Biomarkers of Pathology Severity and Treatment Resistance in Patients With Obsessive‐Compulsive Disorder

**DOI:** 10.1155/da/9694689

**Published:** 2026-02-06

**Authors:** Issa Wassouf, Nicolas Vibert, Julien Dampuré, Damien Doolub, Ghina Harika-Germaneau, Nicolas Langbour, Nematollah Jaafari

**Affiliations:** ^1^ Centre de Recherches sur la Cognition et l’Apprentissage, UMR 7295 CNRS, Université de Poitiers, Université de Tours, Poitiers, Nouvelle-Aquitaine, France, univ-poitiers.fr; ^2^ Unité de Recherche Clinique Pierre Deniker, Centre Hospitalier Henri Laborit, Poitiers, Nouvelle Aquitaine, France; ^3^ Service de Psychiatrie Adulte, Centre Hospitalier du Nord Deux-Sèvres, Thouars, Nouvelle Aquitaine, France; ^4^ Laboratoire de Recherche sur les Processus Psychologiques et Sociaux (2PS), Université Catholique de l’Ouest, Niort, Nouvelle Aquitaine, France; ^5^ Instituto Universitario de Neurociencia (IUNE), Universidad de La Laguna (ULL), La Laguna, Tenerife, Spain, ull.es

**Keywords:** electroencephalography, error-related negativity (ERN), flanker task, obsessive-compulsive disorder, pathology severity, treatment resistance

## Abstract

Obsessive‐compulsive disorder (OCD) is a psychiatric condition that varies considerably in severity and resistance to treatment. The aim of this study was to identify error detection abnormalities in OCD patients using evoked potential recordings and to determine whether links could be established between individual patients’ error detection processes and their severity and resistance to treatment. To answer this question, the potentials evoked by participants’ responses to a flanker task, i.e., the error‐related negativity (ERN/CRN component) and subsequent positivity (Pe/Pc component), were recorded. Twenty‐six OCD patients with a wide range of pathology severity and treatment resistance and 26 control participants matched for gender, age, and education level with the patients were included in the study. The amplitude of the error‐related negativity (ERN) evoked by false responses was positively correlated with the severity of patients’ pathology, while the lower the amplitude of the negativity evoked by correct responses (CRN), the more resistant patients were to treatment. The ERN/CRN components could therefore be used as markers of the severity and treatment resistance of OCD patients’ pathology. Furthermore, under the present experimental conditions, the positive Pe/Pc component, supposed to reflect patients’ awareness of the correctness of their responses, was virtually absent compared to control participants. This suggests a major deficit in the patients’ monitoring of the consequences of their actions. The discovery of this disappearance of action feedback signals in patients leads to proposing an original neurodevelopmental model for the onset of pathology in childhood or adolescence.

## 1. Introduction

Obsessive‐compulsive disorder (OCD) is a psychiatric condition that affects about 2% of the world’s population and is characterized by highly variable levels of severity and resistance to treatment. Executive functions are broadly impaired in patients suffering from OCD [1, 2], and many previous studies using evoked cognitive potentials have focused on the error control process in OCD patients. Indeed, OCD is an illness involving permanent doubt [3], and error‐related potentials were expected to provide insight into the pathophysiological mechanisms underlying the pathology.

The main error‐related potential is the error‐related negativity (ERN), which is evoked in the fronto‐central regions of the scalp when participants make a mistake while responding to a simple forced‐choice task [4] such as a “flanker task” (see description in the Methods section below). The ERN starts just before the incorrect response, reaches its maximum amplitude between 50 and 150 ms after the response, and is thought to be a marker of conflicts between the expected and actual consequences of an action [5].

Many authors have reported that the ERN of OCD patients is greater than that of control participants in a variety of tasks [6, 7]. Whereas many studies have not reported any significant relationship between the amplitude of patients’ ERN and the severity of their pathology, three studies have found that the more severe the pathology, the larger the ERN [8–10]. To the best of our knowledge, the question of whether the ERN and other error‐related evoked potentials differ between treatment‐resistant patients and nonresistant patients has not been evaluated except in one study which, however, involved only three treatment‐resistant OCD patients [11].

Therefore, the main aim of this study was to assess the interindividual variability of error‐related signals in the brains of OCD patients using evoked potential recordings and to evaluate whether the individual characteristics of these potentials could be used as markers of OCD severity and treatment resistance.

### 1.1. Error‐Related Cognitive Potentials and Their Alterations in OCD Patients

In relation to the ERN evoked by erroneous responses, several authors have shown that correct responses to flanker or go no‐go tasks are accompanied by a smaller negative component (CRN) with the same temporal profile as the ERN [12–14]. The ERN/CRN component would therefore be present as soon as participants respond to stimuli, whether or not the response is correct, but would be greater for erroneous responses than for correct ones. Source localization studies of evoked potentials and functional imaging studies indicate that the ERN/CRN component is generated by the anterior cingulate cortex (ACC) [5, 15, 16], which is involved in a wide range of cognitive processes related to cognitive control, such as conflict management, decision‐making, and error processing.

After the ERN/CRN component, the participants’ responses generate a positive component in the centro‐parietal area of the scalp, which occurs around 150–500 ms after the response. This positivity is greater for incorrect responses (Pe component) than for correct responses (Pc component, [4, 17]). Unlike the ERN, the Pe component is only observed for consciously detected errors, not for errors of which the participant is unaware [18, 19]. Source modeling and functional imaging studies indicate that the Pe component is generated by the anterior insular cortex (AIC), a region involved in the processing of salient internal and external events such as consciously perceived errors, but also in interoception and in the integration of sensory and motor information [20–22].

The abnormal increase in ERN amplitude commonly observed in OCD patients compared to control participants confirms that OCD is a pathology characterized by hyperfunction of error control processes, which could be related to the hyperactivity of the ACC that characterizes OCD [6]. Recent studies have shown that the CRN evoked by correct responses is also increased in OCD patients [23, 24], who would thus be faced with a permanent “error signal.” The increased ERN amplitude is also found in patients’ family members unaffected by the pathology and would thus be an endophenotype of the predisposition of families to develop the pathology [25, 26]. In line with this idea, the amplitude of OCD patients’ ERN does not depend significantly on the treatments they receive [27].

According to most authors, the presence of a Pe component when an error is made reflects the participant’s conscious perception of the error [17, 22]. According to Endrass and Ullsperger’s [23] review, the Pe/Pc component would not be altered in OCD patients compared with control participants, which is somewhat surprising, given that most OCD patients have ongoing doubts about whether they have performed particular actions and exhibit checking compulsions. Still, several studies support Endrass and Ullsperger’s assertion [8, 9, 28–30]. In some of them, however, participants received feedback on whether or not their response was correct after each trial [8, 29], which means that they were always aware of their errors and which could explain why the Pe/Pc component was not different between OCD patients and controls in this situation. Indeed, two studies that did not give participants feedback about their responses observed a significant or marginally significant decrease in the amplitude of OCD patients’ Pe component [31, 32].

As in adult OCD patients, children and adolescents with OCD show an increase in ERN amplitude compared to age‐matched control participants. This increase is also seen in siblings not expressing the disease [25, 33–35], confirming that increased ERN amplitude is an endophenotype of a predisposition to develop the pathology. Furthermore, ERN amplitude increases progressively with age in both pediatric OCD patients and age‐matched controls [34, 35]. Interestingly, in pediatric patients, ERN amplitude is negatively correlated with the age of disease onset, meaning that ERN amplitude increased with the duration of the pathology [34, 36].

### 1.2. Predictive Factors and Pathophysiology of Treatment‐Resistant OCD

There is no consensus in the literature regarding the links between treatment resistance and OCD patients’ alterations of cognitive functions [37–41]. Most of these studies only assessed patients’ resistance to a single treatment or set of treatments administered over a relatively short period of time (usually a few weeks) and did not consider all the other treatments patients had received during the course of their illness.

To understand the origin of these discordant results, Doolub et al. [1] explored the links between treatment resistance, executive abilities, and symptom severity in a large sample of patients. In contrast to previous studies, treatment resistance was assessed by considering the clinical impact of all treatments received by patients since the onset of the illness, over a period of up to 30 years, and quantified using Pallanti and Quercioli’s [42] resistance scales. Only performance on one executive function test, the Stroop test, which assesses the ability to inhibit automatic responses, was linked to treatment resistance. Treatment‐resistant patients had lower scores on the Stroop test than others. This result could explain the contradictory findings of previous studies, as it suggests that treatment resistance depends only on one of the components of executive functions defined by Miyake and Friedman [43] and is not related to patients’ performance in tests involving other components.

Using functional imaging, Liu et al. [11] separated three resistant patients from six other patients who responded to drug treatments. The amplitude of the ERN evoked during a flanker task was lower in resistant patients than in responders and tended to be lower than in controls. All patients had longer response times and made more errors than control participants, but treatment‐resistant patients had shorter response times and made more errors than responders. This suggests that the pathophysiology of treatment‐resistant patients may be quite specific and that they may not have an abnormally large ERN like other OCD patients. Shan et al. [44] compared the functional brain connectivity of 20 treatment‐resistant OCD patients and 20 responders. Resistant patients were found to have fewer functional connections in the left middle temporal gyrus than responders, with no significant relationship between this connection density and symptom severity. Interestingly, another study by Fan et al. [45] showed that the patients’ insight into their pathology was positively correlated with the level of spontaneous activity in the left middle temporal gyrus. Finally, Kim et al. [46] showed that OCD patients exhibited greater functional connectivity than controls between the raphe nucleus and temporal cortices, particularly the aforementioned middle temporal gyrus. In patients, the greater this connectivity, the poorer the therapeutic response to selective serotonin reuptake inhibitors (SSRIs). This suggests that left middle temporal gyrus activity and connectivity may be related to both patients’ insight into their disease and treatment resistance.

Other studies have attempted to identify predictors of therapeutic response in OCD patients, but the wide diversity of methodologies and outcomes makes it difficult to identify precise and reproducible markers of resistance [47–53]. Several pathophysiological parameters could predict the variability of treatment responses in OCD patients, but the exact functional links between these abnormalities and the symptoms of the pathology remain elusive.

### 1.3. The Present Experiment

As stated above, the primary goal of this study was to assess whether error‐related evoked potentials could be related to the severity and/or treatment resistance of adult OCD patients’ pathology. The potentials were also compared with those of control participants matched for gender, age, and education level. The data will be discussed in relation with what happens in pediatric patients, which will lead to proposing an original neurodevelopmental model for the onset of OCD during childhood or adolescence.

Consistent with the literature, the ERN was expected to be greater than the CRN (Hypothesis 1 – H1) and the Pe greater than the Pc (H2) in both OCD patients and control participants. In OCD patients, the ERN/CRN amplitudes were expected to be greater than in control participants (H3; [6, 7]), whereas the Pe/Pc amplitudes were expected to be lower than in controls (H4), as previous studies suggest that this component may be of lower amplitude in patients than in controls when participants do not receive feedback about their response [31, 32].

Regarding potential links between error‐related potentials and OCD patients’ clinical variables, a positive correlation was expected between the severity of the pathology and the amplitude of the ERN (H5; [8, 9]). Indeed, the patients tested in the present study presented a wide range of severity levels that should favor the demonstration of this correlation. Treatment‐resistant patients were expected to show alterations of the ERN/CRN component, either in shape or amplitude (H6), compared to other patients [11], and potentially also alterations of the error‐related potentials evoked in the middle temporal gyrus region, whose activity has been linked to treatment resistance ([44, 45]; H7).

In total, the seven experimental hypotheses put forward in this study can be summarized as follows:

H1: ERN should be greater than CRN in both patients and controls.

H2: Pe should be greater than Pc in both patients and controls.

H3: ERN/CRN amplitudes should be greater in patients than in controls.

H4: Pe/Pc amplitudes should be lower in patients than in controls.

H5: There should be a positive correlation between the amplitude of the ERN in patients and the severity of the pathology.

H6: Treatment‐resistant patients should show alterations of the ERN/CRN component compared to other patients.

H7: Treatment‐resistant patients should show alterations in error‐related potentials evoked in the middle temporal gyrus region compared to other patients.

## 2. Material and Methods

### 2.1. Participants

Twenty‐six consecutive patients with a primary diagnosis of OCD (Table 1) were recruited from two psychiatric hospitals within 2 years of a clinical examination by trained psychiatrists. Patients were interviewed according to the Mini International Neuropsychiatric Interview (version 5.0.0, [54]), and their medical records were reviewed to assess any comorbid psychiatric condition. Participants with current or former severe or decompensated mood disorders, schizophrenia, and/or addictive disorders (except tobacco smoking) were excluded. All patients were receiving medication, and 15 of them were undergoing or had undergone neuromodulatory treatment at the time of testing (Table 1).

**Table 1:** Sociodemographic characteristics of the 52 participants included in the analyses and clinical characteristics of the 26 patients (means ± standard deviations).

Sociodemographic characteristics	Patients (*n* = 26)	Controls (*n* = 26)
Age (years)	42.4 ± 14.4 (21–64)	41.9 ± 14.0 (18–62)
Gender (F/M)	15/11	15/11
Number of years of education	12.7 ± 2.4	12.7 ± 2.4
STAI score	52.2 ± 7.0 (38–68)	35.1 ± 6.3 (24–48)**^∗^**
Y‐BOCS score	26.2 ± 7.1 (9–37)	N/A
BABS score (insight)	3.0 ± 2.8 (0–12)	N/A
LNRT score	5.3 ± 3.1 (1–10)	N/A
SRT score	3.4 ± 1.6 (1–7)	N/A
Pharmacological and/or neuromodulatory treatment	26 patients	None
SSRI	26 patients	—
NSRI	3 patients	
Clomipramine	4 patients	
Anxiolytics	14 patients	
Antipsychotics	11 patients	
Neuromodulation by tDCS/rTMS	12 patients	
Neuromodulation by DBS	4 patients	
Cognitive behavioral therapy	12 patients	
Past or current comorbidities	17 patients	None
Bipolarity	2 patients	—
Generalized anxiety disorder	11 patients	
Mild depressive episode	3 patients	
Obs. compulsive personality disorder	2 patients	
Sleep disorders	1 patient	
Tics	3 patients	

*Note:* An asterisk ( ^∗^) indicates a significant difference between patients’ scores and those of matched controls. LNRT, levels of nonresponse to treatments scale; NSRI, serotonin and noradrenaline reuptake inhibitors; SRT, stages of response to treatments scale.

Abbreviations: DBS, deep brain stimulation; N/A, not applicable; rTMS, repetitive transcranial magnetic stimulation; SSRI, selective serotonin reuptake inhibitors; tDCS, transcranial direct current stimulation.

At inclusion, all patients were assessed with the French version of the Yale‐Brown Obsessive‐Compulsive Scale (Y‐BOCS), which measures the severity of OCD symptoms from 0 to 40 [55]. Patients’ level of insight was assessed using their scores on the French version of the Brown Assessment of Beliefs Scale (BABS, [56]), which measures insight using a score ranging from 0 (maximum insight) to 24 (minimum insight). Each participant completed the French adaptation of the State‐Trait Anxiety Inventory (STAI) self‐assessment questionnaire.

The 26 patients were compared with control participants matched to each patient in age (plus or minus 5 years), gender, and education level (plus or minus 1 year), who also completed the STAI questionnaire. The sociodemographic characteristics of patients and controls as well as clinical variables, comorbidities, and current treatments of patients are presented in Table 1. At the time of inclusion, 11 of the patients had a Y‐BOCS score greater than or equal to 30. The state anxiety level was higher in patients than in controls (*t*(41) = 8.29, *p* < 0.001, and Cohen’s *d* = 2.61), consistent with the nature of the OCD pathology.

The study obtained approval from the “*Comité de Protection des Personnes Ile-de France 1*” (CPPIDF1‐2019‐ND29‐cat 2.2) on May 28, 2019. All participants gave written informed consent to participate in the study.

### 2.2. Assessment of Patients’ Resistance to Treatment

As in Doolub et al. [1], patients’ resistance to treatment was assessed by retrospectively considering all pharmacological and psychological treatments received by each patient since the diagnosis of the disease and the start of medical treatment. The characteristics of the pathology, and in particular the types of obsessions and compulsions of each patient, making it possible to determine the dimension(s) of their OCD, were noted on the basis of clinical observations and responses to the Y‐BOCS.

To quantify treatment resistance, we used adapted versions of the two resistance scales designed by Pallanti and Quercioli [42], as in Doolub et al. [1], namely, the “levels of non‐response to treatments” (LNRT) scale and the “stages of response to treatments” (SRT) scale. The LNRT scale includes 10 levels of successive treatments that should be prescribed to OCD patients until a clinical response is observed, in line with the staging of OCD treatments [57–59]. The SRT scale categorizes treatment outcomes into seven levels based on changes in patients’ Y‐BOCS scores [60]. In both cases, higher scores reflect higher treatment resistance.

### 2.3. Experimental Procedure

Participants were seated with their eyes ~60 cm from the computer screen. E‐Prime 2 software (version 2.0.10.356) was used to synchronize EEG recordings with the presentation of visual stimuli and participants’ responses.

**Figure 1:**
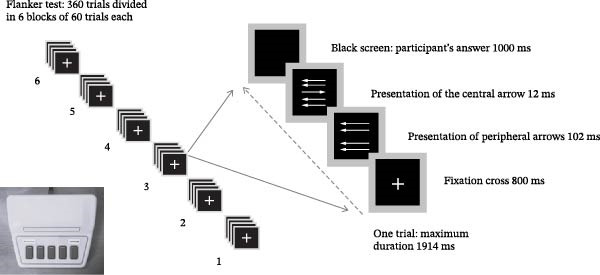
Procedure for the flanker task. In each trial, participants had to press the button corresponding to the direction (left or right) of the central arrow on the response box (photograph in the lower left corner).

In the flanker test, participants saw five horizontal arrows on a computer screen [61] and had to press one of two response buttons with their left or right index fingers, choosing the button corresponding to the direction indicated by the central arrow (Figure 1). On half the trials, the four peripheral arrows pointed in the same direction as the central arrow (“congruent” trials), while on the other half, the peripheral arrows pointed in the opposite direction (“incongruent” trials). The five arrows formed a 1.2 cm square and were presented in white on a black background. Participants had to give their answer within 1 s after the presentation of the central arrow. Participants had to answer as quickly as possible but without making any mistakes. They began with a training block of 60 trials, during which the experimenter checked that the participant understood the written instructions. The test itself comprised 6 blocks of 60 trials. Within each block, half the trials were congruent and the other half incongruent. The central arrow pointed to the left on half the trials and to the right on the other half.

At the end of each block of 60 trials, the software automatically evaluated the participant’s performance. If the subject’s error rate was below 10%, a message was displayed asking him/her to answer faster for the next block. If the error rate was above 30%, the participant was asked to improve his performance. If the error rate was between these two values, the participant was simply asked to continue. The idea was to achieve an error rate between 10% and 30% to obtain an optimal ERN.

### 2.4. EEG Data Recording and Preprocessing

EEG recordings were obtained using a Biosemi Active Two system. EEG traces were recorded at a frequency of 2048 Hz, using a 64‐electrode headset. Four channels were added to monitor eye movements. During recording, the experimenter checked that the data collected were of good quality, i.e., that the electrode impedance was less than five kOhms.

EEG traces were analyzed using Brain Vision Analyzer 2 software (Version 2.01.391, Brain Products) and Matlab R2008b software (Mathworks). The EEG was preprocessed as follows:

• Application of a bandpass filter between 0.1 and 30 Hz (24 dB per octave).
• Calculation of an average reference over all the electrodes and rejection by the software of the recording segments presenting artifacts or poor‐quality tracings.
• Offline correction of eye movement artifacts using the Gratton and Coles algorithm [62].

The time window for the analysis of error‐related evoked potentials ranged from 200 ms before the participant’s response to 500 ms afterwards, with zero set at the moment the response button was pressed. The 200 ms of recording before the response was used to calculate the mean potential baseline. The EEG data windows thus created for each trial were divided into two categories according to whether the response was correct or incorrect, while nonresponses were not taken into account. The potentials evoked by correct and incorrect responses were then averaged separately for each electrode and participant.

For correct responses, the software’s rejection rate for poor‐quality recording segments was 8.4% for patients and 0.7% for controls, while for incorrect responses, the rejection rate was 15.4% for patients and 10.3% for controls. In all cases, this rate remained well below the commonly accepted limit of 20%, ensuring quality recordings.

### 2.5. Statistical Analysis of the Data

All statistical analyses were carried out using Statistica software (version 14.0.0.15), with a significance threshold set at 5%. The numbers of incorrect responses and nonresponses given by participants, as well as their response times for correct responses, were subjected to ANOVAs using pathology (patients versus controls) and stimulus type (congruent versus incongruent) as factors. Effect sizes were given by partial eta squares (eta^2^_P_). For OCD patients, correlation analyses were also conducted to determine whether these behavioral measures were related to pathology severity, insight, and/or treatment resistance.

To analyze the potentials evoked by participants’ responses, the first step was to quantify their amplitudes. Following preprocessing of the EEG recordings, the mean potentials evoked by the correct and incorrect responses of all participants were visually examined. This examination made it possible to identify two “time windows” during which tracings appeared to differ between patients and control participants and/or between incorrect and correct responses.

• The first window corresponded to a negative component maximal in fronto‐central areas, starting 20 ms before the response, peaking 40 ms after, and ending 100–120 ms after the response, identified as the ERN/CRN.
• The second window corresponded to a positive component also maximal in the fronto‐central areas, which began 100–120 ms after the response, peaked 150–200 ms after, and ended around 400 ms after the response, identified as the Pe/Pc.

The amplitudes of these two components were measured by quantifying the area between the mean EEG trace and the 0 potential (*x*‐axis line) between 20 and 60 ms after the participants’ response for the ERN/CRN and between 170 and 270 ms after the response for the Pe/Pc.

**Figure 2:**
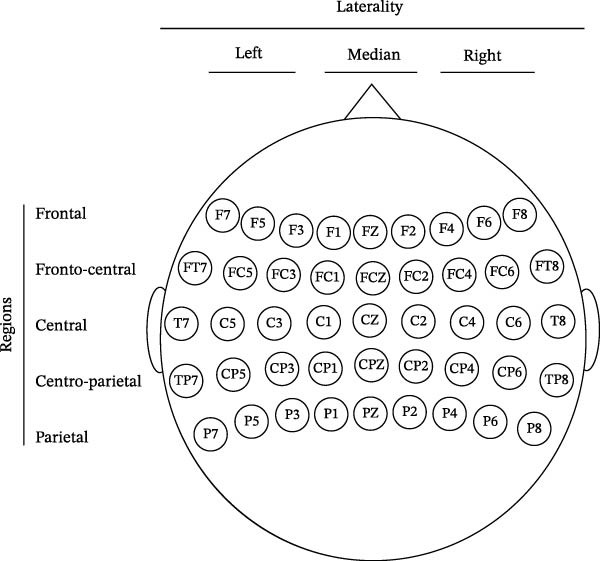
Representation of the distribution of electrodes on the scalp and of the topographical variables used to study the ERN/CRN and Pe/Pc components. In the coronal plane, the frontal region includes electrodes F7, F5, F3, F1, Fz, F2, F4, F6, and F8; the fronto‐central region includes electrodes FT7, FC5, FC3, FC1, FCz, FC2, FC4, FC6, and FT8; the central region includes electrodes T7, C5, C3, C1, Cz, C2, C4, C6, and T8; the centro‐parietal region includes electrodes TP7, CP5, CP3, CP1, CPz, CP2, CP4, CP6, and TP8; and the parietal region includes electrodes P7, P5, P3, P1, Pz, P2, P4, P6, and P8. In the sagittal plane, the left laterality includes electrodes F3, FC3, C3, CP3, P3, F5, FC5, C5, CP5, P5, F7, FT7, T7, TP7, and P7; the median laterality includes the central and paracentral electrodes Fz, FCz, Cz, CPz, Pz, F1, FC1, C1, CP1, P1, F2, FC2, C2, CP2, and P2; and the right laterality includes electrodes F4, FC4, C4, CP4, P4, F6, FC6, C6, CP6, P6, F8, FT8, T8, TP8, and P8.

These amplitudes, obtained for each electrode, were analyzed using ANOVAs with Pathology (patients versus controls), Response type (incorrect or correct), Regions, which designate the five different coronal planes defined from front to back, each comprising nine electrodes (Figure 2), Laterality, which designates the three different sagittal planes defined from left to right, each comprising 15 electrodes (Figure 2), and Electrode with three modalities, each modality respectively grouping the left, central, or right electrodes of all 15 cerebral areas defined by the intersection of the five regions and the three lateralities (Figure 2). Pillai correction was applied for the four intraparticipant factors. Follow‐up two‐way comparisons were performed using planned comparisons.

For patients, additional analyses were performed, adding measures of disease severity, insight, and treatment resistance as covariates. When a significant correlation was observed between these clinical variables and the amplitudes of the response‐evoked potentials, correlational analyses were used to determine the electrodes and brain areas where this correlation was significant.

## 3. Results

### 3.1. Behavioral Results

Table 2 presents the response times of patients and controls on the flanker test and the number of errors (incorrect responses and nonresponses) they made for congruent (180 trials) and incongruent trials (180 trials). Patients’ response times and error rates did not depend significantly on the type of treatment they were receiving or their comorbidities.

**Table 2:** Number of errors and response times of OCD patients and control participants in the flanker test (means ± standard deviations).

Nature of trials	Patients		Controls	
	Congruent	Incongruent	Congruent	Incongruent
Response time (ms)				
Correct answers	435 ± 109	491 ± 120	415 ± 63	465 ± 70
Incorrect answers	533 ± 180	415 ± 134	561 ± 117	419 ± 128
Number of errors	16.5 ± 15.7	59.8 ± 53.7	9.5 ± 7.9	32.1 ± 29.4
Error rate	9.2%	33.2%	5.3%	17.8%

For correct responses, reaction times were not significantly different between patients and controls (*F*(1,50) = 0.83 and *p* = 0.37). In contrast, as expected, all participants’ responses were longer when stimuli were incongruent than when stimuli were congruent (*F*(1,50) = 56.03, *p* < 0.001 and *ƞ* ^2^_p_ = 0.53). Patients made more errors than controls, whether stimuli were congruent or incongruent (*F*(1,50) = 6.48, *p* < 0.05, and *ƞ* ^2^_p_ = 0.11), and all participants made more errors on incongruent stimuli than on congruent stimuli (*F*(1,50) = 34.27, *p* < 0.001, and *ƞ* ^2^_p_ = 0.41). No significant correlation was observed between the anxiety level of patients (−0.17 < all *r*s < 0.20; all *p*s > 0.37) and controls (−0.28 < all *rs* < 0.09; all *ps* > 0.25) and their response times or number of errors. Among patients, there was also no significant correlation between pathology severity, insight, or treatment resistance scores and response times or number of errors (−0.22 < all *rs* < 0.39; all *ps* > 0.06).

### 3.2. Results of Electroencephalographic Recordings

The amplitude of the ERN/CRN and Pe/Pc components of the patients’ evoked potentials did not depend significantly on the type of treatment they were receiving or their comorbidities. To investigate whether differences in the preresponse baseline potentials used as a reference for measuring the ERN/CRN and Pe/Pc components could explain some of the results described below for these components, the amplitudes of the potentials evoked by the appearance of the central arrow of the flanker stimuli in this time window (~300–500 ms poststimulus) were measured and then analyzed in the same way as the response‐evoked potentials. None of the significant differences, correlations, or interactions identified and analyzed below for either the ERN/CRN or the Pe/Pc components were significant within the time window used as a baseline for these potentials. This suggests that none of our data were due to significant differences in the baselines used as a reference to measure the ERN/CRN and Pe/Pc components.

#### 3.2.1. ERN/CRN Component (20 to 60 ms)

**Figure 3:**
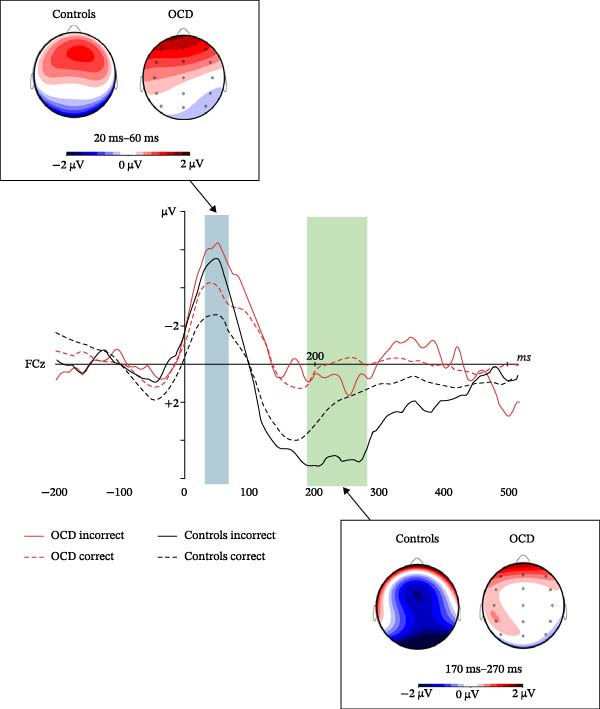
Magnifications of the potentials evoked by correct and incorrect responses at the FCz electrode and scalp distribution of electrical activity corresponding to the difference between incorrect and correct responses in patients and controls. Blue and green areas indicate the time windows used to analyze the amplitudes of the ERN/CRN and Pe/Pc component, respectively, in the two groups of participants. The top left and bottom right panels show the distribution of electrical activity corresponding to the difference between incorrect and correct responses for the ERN/CRN (20–60 ms time window) and Pe/Pc (170–270 ms time window) components.

Although the amplitudes of the ERN and CRN appeared to be higher in patients than in controls (Figure 3), the ANOVA did not reveal a significant effect of pathology on the amplitude of this component (*F*(1,50) = 0.42, *p* = 0.52), mainly due to the very high variability of amplitudes within each group of participants. There was also no significant interaction between pathology and the other factors (all *Fs* < 1.99 and all *ps* > 0.14). However, the ANOVA revealed an interaction between region and response type (*F*(4,47) = 3.43, *p* < 0.05, and *ƞ* ^2^_p_ = 0.23). Follow‐up comparisons showed that for patients and controls, the ERN evoked by incorrect responses was larger than the CRN evoked by correct responses (Figure 3) in the frontal (−1.50 ± 1.84 µV versus −0.95 ± 1.67 µV, *F*(1,50) = 3.96, and *p* = 0.05) and fronto‐central regions (−1.2 ± 1.44 µV versus −0.78 ± 1.17 µV, *F*(1,50) = 8.27, and *p* < 0.01) but not in the central, centro‐parietal, or parietal regions (all *Fs* < 3.03 and all *ps* > 0.08).

Additional analyses revealed a positive correlation between the ERN/CRN amplitude and pathology severity as estimated by the Y‐BOCS score (*F*(1,24) = 5.03, *p* < 0.05, and *ƞ* ^2^_*p*_ = 0.17), modulated by an interaction between pathology severity and region (*F*(4,21) = 3.22, *p* < 0.05, and *ƞ* ^2^_*p*_ = 0.38), and a three‐way interaction between pathology severity, region, and response type (*F*(4,21) = 2.93, *p* < 0.05, and *ƞ* ^2^_*p*_ = 0.36). In general, the more negative the ERN/CRN, the more severe the pathology (correlation coefficient *r* = −0.42 and *p* < 0.05). Decomposition of the triple interaction by response type showed that the interaction between region and pathology severity was significant only for the ERN (*F*(4,21) = 5.17, *p* < 0.01, and *ƞ* ^2^_p_ = 0.50) but not for the CRN (*F*(4,21) = 0.90 and *p* = 0.48). ERN amplitude was greater the more severe the pathology was in the central (*r* = 0.42 and *p* < 0.05), centro‐parietal (*r* = 0.55 and *p* < 0.01), and parietal (*r* = 0.52 and *p* < 0.01) regions but not in the frontal (*r* = −0.34 and *p* = 0.09) and fronto‐central (*r* = 0.11 and *p* = 0.60) regions.

**Figure 4:**
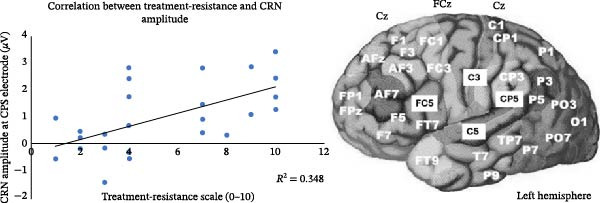
Positive correlation between patients’ LNRT scores and CRN amplitude. The higher the LNRT score at electrodes FC5, C5, C3, and CP5, the less negative the CRN. As treatment resistance increases, CRN amplitude decreases at the temporo‐parieto‐frontal junction (right panel). The left panel shows the positive correlation between the potential obtained at CP5 during the temporal window of the CRN and the level of nonresponse to treatment (LNRT) for the 26 patients included in the experiment.

Further analysis also revealed that the impact of patients’ LNRT scale score interacted with response type (*F*(1,24) = 6.27, *p* < 0.05, and *ƞ* ^2^_*p*_ = 0.21). CRN amplitude, but not ERN amplitude, was positively correlated with the patients’ LNRT scores (*r* = 0.42 and *p* < 0.05), in other words, the more treatment‐resistant patients were, the less negative their CRN was. This correlation was significant for electrodes FC 5 (*r* = −0.45 and *p* < 0.05), C5 (*r* = −0.56 and *p* < 0.01), C3 (*r* = −0.49 and *p* < 0.05), and CP5 (*r* = −0.59 and *p* < 0.01), that is, at the level of the left temporo‐parieto‐frontal junction (Figure 4).

#### 3.2.2. Pe/Pc Component (170 to 270 ms)

There was not any significant main effect of pathology (*F*(1,50) = 0.30 and *p* = 0.59) or response type (*F*(1,50) = 0.17 and *p* = 0.68) on the amplitude of the Pe/Pc component (Figure 3), but there were several interactions between region and pathology (*F*(4,47) = 3.09, *p* < 0.05, and *ƞ* ^2^_*p*_ = 0.21); between region and response type (*F*(4,47) = 7.77, *p* < 0.001, and *ƞ* ^2^_*p*_ = 0.40); between laterality and response type (*F*(2,49) = 6.95, *p* < 0.01, and *ƞ* ^2^_*p*_ = 0.22); between laterality, response type, and pathology (*F*(2,49) = 4.81, *p* < 0.05, and *ƞ* ^2^_*p*_ = 0.16); between region, laterality, and response type (*F*(8,43) = 4.77, *p* < 0.001, and *ƞ* ^2^_*p*_ = 0.47); and between laterality, electrode, response type, and pathology (*F*(4,47) = 4.09, *p* < 0.01, and *ƞ* ^2^_*p*_ = 0.26). Planned comparisons related to the interaction between region and pathology showed that both Pe and Pc components were larger in controls than in patients over the entire central region (0.93 ± 1.21 µV versus 0.14 ± 1.21 µV, *F*(1,50) = 5.56, and *p* < 0.05) but not over other regions (all *Fs* < 2.31 and all *ps* > 0.13).

In view of the graphs obtained (Figure 3) and our hypotheses, the most interesting interaction to analyze was that between laterality, response type, and pathology. Its decomposition according to response type indicated that the interaction between pathology and laterality was significant only for the Pe component (*F*(2,49) = 4.40, *p* < 0.05, and *ƞ* ^2^_*p*_ = 0.15) but not for the Pc component (*F*(2,49) = 0.88 and *p* = 0.42). Planned comparisons indicated that the Pe was more positive in controls than in patients only in the median laterality (1.54 ± 1.85 µV versus 0.29 ± 1, 85 µV, *F*(1,50) = 5.96, and *p* < 0.05) but not in the left (−0.62 ± 1.50 µV versus 0.13 ± 1.50 µV, *F*(1,50) = 3.18, and *p* = 0.08) or right laterality (−0.14 ± 1.72 µV versus 0.15 ± 1.72 µV, *F*(1,25) = 0.35, and *p* = 0.56). Decomposition of the same interaction by pathology showed that the interaction between laterality and response type was significant only for control participants (*F*(2,24) = 10.42, *p* < 0.001, and *ƞ* ^2^_*p*_ = 0.46) and not for patients (*F*(2,24) = 0.49 and *p* = 0.62). In control participants, the Pe component was larger or marginally larger than the Pc component in the left (0.05 ± 1.11 µV versus −0.62 ± 1.37 µV, *F*(1,25) = 3.41, and *p* = 0.08) and median laterality (1.54 ± 1.75 µV versus 0.45 ± 1.34 µV, *F*(1,25) = 12.64, and *p* < 0.01) but not in the right laterality (−0.14 ± 1.17 µV versus 0.28 ± 1.89 µV, *F*(1,25) = 2.25, and *p* = 0.15).

To summarize (see the potentials evoked at the FCz electrode illustrated in Figure 3), in the median sagittal part of the scalp, the Pe was larger in controls than in patients, whereas the Pc amplitude was not significantly different between patients and controls. In the same median part of the scalp, the Pe was larger than the Pc in control participants but not in patients, in whom Pe and Pc components were virtually absent. Consequently, both Pe and Pc were larger in controls than in patients in the central region, regardless of laterality. Taken together, this shows that at the scalp apex, which corresponds to the intersection between the central and fronto‐central regions and the median sagittal part of the scalp (electrodes Cz and FCz, for instance), the Pe/Pc component was virtually absent in patients, whereas it was present in control participants, in whom the Pe evoked by incorrect responses was, as expected, larger than the Pc evoked by correct responses.

## 4. Discussion

### 4.1. Behavioral Data

In the flanker task, reaction times of OCD patients and controls were longer for incongruent stimuli than for congruent stimuli, and both groups made more errors in response to incongruent stimuli. Thus, consistent with the literature, distracting arrows caused interference when they pointed in the opposite direction to the central arrow. Also consistent with the literature, there was no significant difference between reaction times of patients and controls. However, patients made more errors than controls, an inconsistent finding in the literature, which may be related to methodological differences such as stimulus presentation rates. Indeed, OCD patients appear to make more errors than controls in the flanker task when the central arrow is presented for very short times (10–12 ms) as in the present study [11] but not when the central arrow is displayed for longer times (30–50 ms, [8, 9, 32]).

### 4.2. Electroencephalographic Data

#### 4.2.1. ERN/CRN Component

As in previous studies, and in agreement with H1, the ERN evoked by incorrect responses was larger than the CRN evoked by correct responses in both OCD patients and controls and in frontal and fronto‐central regions. However, contrary to expectations, H3 was not verified, as the patients’ ERN and CRN were not significantly larger than those of controls. Although these two components appear larger in patients than in the control in Figure 2, the difference was not statistically significant due to large interindividual variability.

The lack of significant increase in ERN/CRN amplitude in OCD patients compared with controls, despite a clear trend for the amplitudes of the ERN and CRN to be higher in patients (see Figure 3) contradicts a fairly unanimous previous literature (see [7]) but could be related to the specific characteristics of the patient sample. Indeed, the patients included in this study were on average more severely affected than in previous studies. Their mean Y‐BOCS score was over 26, and almost half of patients were resistant or very resistant to treatment (Table 1). In comparison, patients included in previous studies that showed a significant increase in the ERN/CRN amplitude of OCD patients compared with controls [8, 9, 26, 28–30, 32] had significantly less severe forms of the disorder, with mean Y‐BOCS scores ranging from 17 to 22.5. Moreover, most of these studies did not address the notion of treatment resistance, and the only experiment that did look at treatment resistance [11] suggested that resistant patients present a less ample ERN than responders. Thus, the lack of difference in ERN/CRN amplitude between OCD patients and controls may result from the fact that in the present study, almost half the patients were resistant to treatment.

Hypothesis H5, which postulated a positive relationship between ERN amplitude and pathology severity, was verified. This result, which confirms the data obtained by Endrass et al. [8, 9], was probably made more visible by the wide variation in disease severity among the patients included in the present study. However, contrary to what was found by Endrass et al., this correlation was only significant in the central, centro‐parietal, and parietal regions and not in the frontal or fronto‐central regions where ERN is maximal. This somewhat unexpected localization of the correlation with pathology severity may explain why many authors did not find it, as they only examined error‐evoked potentials in anterior scalp regions [26, 29, 30]. This could also explain why, despite observing this correlation in certain areas of the scalp, the overall amplitude of ERN, assessed across the entire scalp, did not differ significantly between patients and controls. Furthermore, the existence of this correlation suggests considerable variability in ERN amplitude in the areas of the scalp where it was observed, which could also explain the lack of significant difference in ERN amplitude between patients and controls.

With regard to treatment resistance, both H6 and H7 were verified. For the first time, a link was found between one of the treatment resistance scales, that of the LNRT, and the amplitude of the CRN evoked in the area of the left temporo‐parieto‐frontal junction (Figure 4). These potentials may be related to the activation of neurons in the left middle temporal gyrus (at electrodes T7 and TP7), where functional imaging has shown an association between brain activity and treatment resistance in OCD patients [44, 45]. However, the direction of this correlation was somewhat unexpected and remains difficult to interpret. Indeed, the more resistant patients were to treatment, the less negative the CRN obtained at electrodes C3, C5, FC5, and CP5 was, which seems to contradict the idea that the most resistant patients are also those with the most severe symptoms (see [1]) and therefore the highest amplitude ERN/CRN component. Nevertheless, this finding appears to be consistent with the report by Liu et al. [11], which found a weaker ERN in highly resistant patients than in other patients.

#### 4.2.2. Pe/Pc Component

In the medial sagittal part of the scalp, where the Pe/Pc component is normally maximal, this component was virtually absent in patients, whereas it was observed in controls. This also implies that in patients, there was no significant difference between the Pe and Pc. Consequently, H2 was verified only in control participants, in whom the Pe component was, as usual, greater than the Pc component. The decrease of the Pe/Pc in OCD patients is in line with H4, which postulated a smaller Pe/Pc in patients than in controls, but the virtual disappearance of this component in patients was unexpected. In line with the idea that the Pe component represents the error‐maker’s awareness of the error [18, 19, 63], this result strongly suggests a total absence or strong reduction of the feedback that distinguishes correct from false responses in patients in the conditions of this experiment.

Such a large difference between the Pe/Pc of OCD patients and controls has not yet been reported in the literature, although two studies have already shown a decrease in Pe amplitude in patients compared with controls [23, 32]. Several reasons may explain this inconsistency. Firstly, the flanker task used in this study involved trials in faster succession than in previous studies. Indeed, the maximum duration of each trial was 1914 ms in this study (Figure 1) compared to the average duration of 2200 to 3700 ms used in most former studies of error‐related potentials in OCD patients [8, 9, 23, 28, 29, 32]. Secondly, the task did not include any explicit feedback about response accuracy such as for example in Endrass et al. [8, 9], Liu et al. [11], and Ruchsow et al. [29]. Thirdly, patients included in this study were on average more severely affected and more resistant to treatment than in previous studies (Table 1). This latter idea that the amplitude of OCD patients’ Pe/Pc may be lower in more severely affected and/or more resistant patients is supported by a careful review of previously published articles using the flanker task, which shows that studies in which patients’ mean Y‐BOCS score was between 17 and 20.5 [8, 9, 29, 30] found no significant differences in Pe amplitude between patients and controls. In contrast, two studies in patients with more severe pathology (mean Y‐BOCS score between 22 and 23; [31, 32]) concluded that OCD patients’ Pe tended to be smaller than in controls at FCz, which is closer to the current findings.

To assess whether a similar decrease of the Pe/Pc component could be observed in pediatric OCD patients, the authors of the present study reviewed the papers that have explored error‐related evoked potentials in this population or in children exhibiting obsessive‐compulsive behaviors, as assessed by their parents. In most cases, only the amplitude of the ERN/CRN component was measured and statistically analyzed. However, close examination of the Pe/Pc tracings obtained in these studies revealed that in most of them, the amplitude of the Pe component appeared to be substantially smaller in children with OCD than in control children or adolescents [25, 33–36, 64, 65]. Moreover, this apparent decrease in Pe amplitude was not observed in unaffected siblings [25]. In contrast to these data, Santesso et al. [66] found that in 10‐year‐old children, more pronounced obsessive‐compulsive behaviors were associated with larger ERN and Pe components during a visual flanker task. However, this result was obtained in a population of typical, nonpathological children, and the amplitudes of the ERN and Pe were also inversely correlated with the number of errors made by the children during the task, suggesting that their increase could result from personality traits inducing heightened vigilance and/or caution, potentially influencing both the error rate and evoked potentials. Recently, Hanna et al. [67] found that the difference between the amplitudes of the Pe and Pc components was significantly smaller in children with OCD than in age‐matched controls. Altogether, this suggests that the sharp decrease in the amplitude of the Pe/Pc component and of the difference between the amplitudes of these two components seen in adult OCD patients in the present study may also be observed in young children and adolescents with this condition.

### 4.3. Consequences of the Decrease in the Amplitude of the Pe/Pc Component in OCD Patients

As previously indicated, this experiment was designed to have a very rapid succession of trials, with the idea that participants would have fewer opportunities to evaluate the quality of their responses than in previous studies. In this context, the near disappearance of the Pe/Pc component in OCD patients suggests that they were forced to give their answers without any feedback on their accuracy, whereas the controls were aware of their errors, perhaps because the feedback signals would be generated more quickly and efficiently in their case.

To a certain extent, it can therefore be said that the present experimental conditions highlighted an alteration in the patients’ awareness of errors, or more generally of the actions performed, which could concern all the simple gestures usually performed without thinking in everyday life (locking or not locking a door, turning off or not the gas or electricity, etc.). Indeed, while the expected responses in the flanker task cannot be given without reflection but rather require inhibitory control over distracting elements, they share with these simple gestures their simplicity and repetitiveness. This would generate, in both cases, the same kind of uncertainty in OCD patients regarding the correct execution of the required action. This altered awareness of their actions could explain the tendency towards incessant checking that affects a large proportion of them and more generally lead to the alterations in metacognition that characterize the disorder. Indeed, according to some authors, the absence of an effective error detection system and the impaired ability of the ACC to integrate the emotional and cognitive signals monitoring the consequences of actions could be the main driving force behind the onset of pathology [68, 69].

The mechanism behind the onset of the pathology may be summarized as follows. Most OCD patients recognize the absurdity of their obsessions and compulsions and their detrimental impact on their daily lives. This suggests that the impairment of patients’ metacognition remains limited to the simple gestures of daily life most often performed without thinking. However, OCD not only gives rise to repetitive motor actions such as checking, washing, or tidying up but also to unacceptable thoughts, often so absurd that they do not lead to feasible actions but generate mental compulsions. What these mental compulsions have in common with the pathological repetition of simple actions is that they neutralize the permanent anxiety felt by patients by performing a physical or mental act with full awareness and without uncertainty, which temporarily eliminates this anxiety. Unfortunately, the incessant repetition of these acts and the absence of clear feedback on whether or not the act has been performed would then very quickly prevent the patient from knowing whether or not the act has actually been carried out [70]. This detour of simple mental or motor acts aimed at neutralizing anxiety, and the resulting uncertainty about whether these acts were actually performed, would underlie the dysfunctional beliefs of OCD patients, and in particular the abnormal metacognitive beliefs that lead patients to believe that thinking about an event increases the likelihood that it will occur, and/or that thinking about doing an action increases the likelihood that they will perform it even if they do not want to (see [3] for a recent review).

### 4.4. Towards a Neurodevelopmental Model of the Appearance of OCD

**Figure 5:**
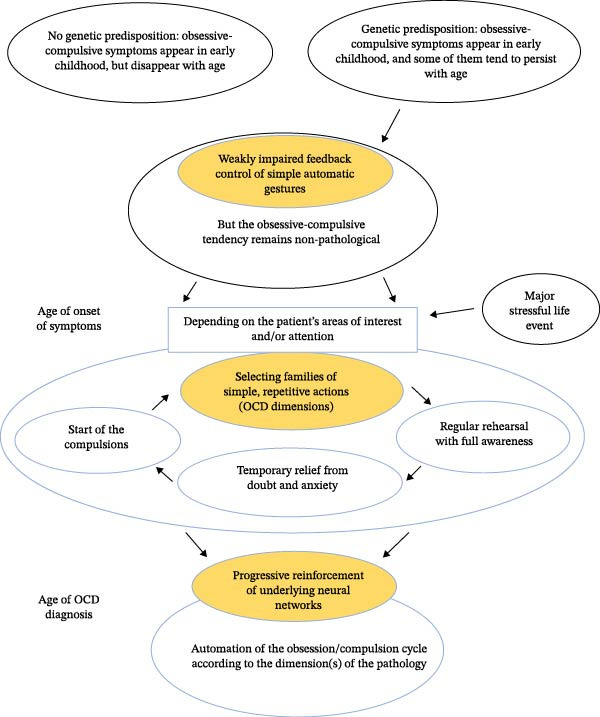
Proposal of a neurodevelopmental model for obsessive‐compulsive disorder. In the absence of genetic predisposition, the obsessions and compulsions commonly observed at the age of 4 or 5 gradually disappear. However, if action feedback signals are abnormally weak, children will continue to repeat certain simple gestures from time to time, without the intensity of the repetitions being pathological. But if these children with weak feedback control signals are then subjected to a stressful life event, this event may trigger the onset of the first obsessive‐compulsive symptoms of pathological intensity, the dimensions of which are determined by each person’s own focus of interest or attention. The disease is then progressively consolidated through the repetition of obsessive‐compulsive cycles, in a vicious circle that gradually reinforces the underlying neural networks and leads to a loss of control through the automation of obsessive‐compulsive sequences. OCD is generally diagnosed only when this automatization is already well advanced and established.

In view of the results of the present study and data from studies carried out in children, we suggest that the pathophysiological mechanism underlying the pathology is primarily an alteration, evolving from childhood, in the feedback signals that control the consequences of simple and automatic gestures. This hypothesis enables us to propose a comprehensive neurodevelopmental model of the onset and development of the pathology with age, as described in Figure 5. These simple gestures, which are performed without thinking, are widely present in our daily lives, and their effortless execution is essential if we are to direct our attention and use our executive capacities to plan the realization of more complex goals. Indeed, these simple gestures are often integrated into a series of actions aimed at a more general goal, such as getting to work (closing the door on leaving) or cooking (turning off the gas stove). So, although these gestures are performed largely automatically, the executive control system needs to be informed of their correct execution by a feedback signal so that the more complex task in which these gestures form part can proceed smoothly. If the feedback signal constituted by the Pe/Pc component disappears, as was the case with patients under the conditions of this experiment, this lack of information from the executive control system will be a source of anxiety for patients and of doubts about the overall quality of their own cognition.

Following this line of thinking, young patients could present the first symptoms of the pathology as soon as they begin to automate the simple gestures of daily life discussed above, around the age of 4 or 5 years, the age at which most children do present repetitive and ritualized behaviors (see [71] for a recent review). Normally, these behaviors disappear with age, but if, for whatever reason (probably some genetic background), the signals indicating their correct execution are a little weak, some children may continue to tend to repeat certain simple gestures of their daily life in full awareness, with slow, controlled movements so as not to have any doubts about their correct execution. It is therefore possible that, particularly under the pressure of a stressful event in their environment, during childhood or adolescence, or even sometimes later, some children with deficits in Pe/Pc feedback signals may start repeating these gestures abnormally often to eliminate the general anxiety they are otherwise feeling, which would be the beginning of compulsions and intrusive thoughts of not having carried out the intended action. Indeed, a recent systematic review by Baldini et al. [72] on the impact of childhood trauma on OCD concludes that individuals with OCD frequently experienced childhood trauma and that emotional abuse and neglect are significantly associated with higher Y‐BOCS scores. According to Raposo‐Lima and Morgado [73], it seems reasonable to acknowledge the existence of a gene–environment interaction in the pathophysiology of OCD, as the influence of various stressful events or environmental aggressions has been widely described as influencing the natural course of the disorder and increasing the risk conferred by genetic inheritance.

The scope and nature of compulsions and intrusive thoughts would be related to each patient’s personal experience, perhaps also to the nature of the stressful event that triggered them, and would also be a function of their particular interests. According to Salkovskis [74], obsessions begin to emerge with the spontaneous appearance of intrusive thoughts. Although such intrusive thoughts exist in typical individuals, in patients with OCD, they are subject to interpretation that is embedded in pathological thought patterns and dysfunctional beliefs [74]. These thought patterns give OCD its pathological character by transforming “ordinary” intrusive thoughts into obsessions. Because several years often elapse between the onset of these symptoms and the diagnosis, the repetition of obsessive/compulsive cycles throughout this period would lead to a progressive reinforcement and functional hyperactivation of brain networks linked to performance control, particularly in patients’ areas of interest, which would determine the dimension(s) of the pathology. In the model presented here, intrusive thoughts and obsessions would be the result of the hyperfunctionality of these networks. Ultimately, failure to take early action against symptoms leads to their progressive worsening over the years. Unfortunately, most patients seek help only late in life, when symptoms have already become incapacitating.

### 4.5. Conclusion and Limitations of the Study

All in all, the data from the present study and other studies of error‐related electroencephalographic potentials in children and adults suggest a major deficit in patients’ monitoring of the consequences of their actions. The model presented in Figure 5 therefore proposes that the anxiety‐inducing doubt linked to a genetic weakness in action feedback signals, associated with a stressful event in the patient’s environment, would be the trigger for the mechanism that sets up and progressively reinforces OCD.

This study has several limitations that should be acknowledged for transparency. First, the neuromodulatory treatments (i.e., tDCS, rTMS and/or DBS, see Table 1) received by 15 of the patients were highly diverse, especially since the cortical or subcortical structures targeted by these treatments varied from one patient to another. As indicated in the results section, neither the patients’ response times and error rates nor the amplitude of the ERN/CRN and Pe/Pc components of the patients’ evoked potentials depended significantly on the type of treatment received. However, this lack of consistency of neuromodulatory treatments across patients may have masked potential effects of some of these treatments on OCD patients’ error‐related evoked potentials. This issue should be addressed in a study specifically designed to assess more precisely the impact of these different neuromodulation techniques on these potentials.

Second, the relatively small number of patients included in the study limited the power of the correlational analyses conducted to assess the impact of the severity of the patients’ pathology and their treatment‐resistance scores on their behavioral responses and error‐related potentials. The conclusions of these correlational analyses should therefore be viewed with caution.

Third, some results of the present study differed somewhat from those reported in previous literature, notably the absence of increase in ERN/CRN amplitude in OCD patients compared with controls and the almost complete disappearance of the Pe/Pc component in OCD patients. Several factors could explain these differences, such as the rapid succession of trials compared to most previous studies and the fact that the patients included in this study were on average more severely affected and more resistant to treatment than in previous studies. However, further studies more directly evaluating the impact of these factors on error‐related potentials are needed to confirm these findings. In particular, studies specifically focusing on the impact of treatment resistance on error‐related potentials, using larger samples of treatment‐resistant patients compared to nonresistant patients, should be conducted.

## Author Contributions

**Issa Wassouf and Nicolas Vibert:** article writing, protocols, design set‐up, proof reading, data analysis, data interpretation, article submission. **Julien Dampuré:** protocols, design set‐up, proof reading, data analysis, data interpretation. **Damien Doolub:** proofreading, advisor. **Ghina Harika-Germaneau:** proofreading, advisor. **Nicolas Langbour:** protocols, design set‐up, proof reading. **Nematollah Jaafari:** investigator, proofreading, advisor.

## Funding

This research did not receive any grant from funding agencies in the public, commercial, or not‐for‐profit sectors.

## Conflicts of Interest

The authors declare no conflicts of interest.

## Data Availability

The data that support the findings of this study are available from the corresponding author upon reasonable request.
